# High-risk Therapeutic Devices Approved by the US Food and Drug Administration for Use in Children and Adolescents From 2016 to 2021

**DOI:** 10.1001/jamapediatrics.2022.4131

**Published:** 2022-11-07

**Authors:** Kavya Pathak, Claire Narang, Thomas J. Hwang, Juan C. Espinoza, Florence T. Bourgeois

**Affiliations:** 1 Harvard Medical School, Boston, Massachusetts; 2 Computational Health Informatics Program, Boston Children’s Hospital, Boston, Massachusetts; 3 Division of Urological Surgery, Department of Surgery, Brigham and Women’s Hospital, Boston, Massachusetts; 4 Department of Pediatrics, Children’s Hospital Los Angeles, Los Angeles, California; 5 Department of Pediatrics, Harvard Medical School, Boston, Massachusetts

## Abstract

This cohort study examines the characteristics of high-risk therapeutic devices approved by the US Food and Drug Administration for use in children and adolescents between 2016 and 2021.

Few medical devices are developed specifically for use in children, although pediatric patients have unique requirements related to device indication and function. Thus, physicians often repurpose or modify devices designed for adults to meet pediatric needs.^1^ In most cases, data on safety and effectiveness for these off-label applications are lacking.^2^ Congress and the US Food and Drug Administration (FDA) have introduced several legislative and regulatory strategies, including the Pediatric Medical Device Safety and Improvement Act (passed in 2007), to expand pediatric-specific medical device development.^3,4^ The aim of this study was to evaluate and compare characteristics and premarket and postmarket clinical evidence for FDA-approved high-risk therapeutic devices for pediatric and adult populations.

## Methods

In this cohort study, we queried the FDA’s Premarket Approval^5^ and Humanitarian Device Exemption^6^ databases to identify all high-risk (class III) therapeutic medical devices with first-time approvals between January 1, 2016, and December 31, 2021. We abstracted regulatory and therapeutic characteristics and classified devices as approved for children (aged <18 years) or adults (aged ≥18 years) (eTable in the Supplement). The Boston Children’s Hospital Institutional Review Board deemed the study exempt from review and waived the requirement for informed consent because it relied on publicly available, nonclinical data. We followed the STROBE reporting guideline.

Information on pivotal studies supporting FDA approval and on required postapproval studies were obtained from the previously mentioned FDA databases and from ClinicalTrials.gov, with follow-up through February 14, 2022. Participants’ ages were identified based on trial eligibility criteria and trial results describing participant ages.

We performed descriptive statistics to compare pediatric and adult devices and clinical studies using Microsoft Excel, version 2206. A 2-tailed *P* < .05 was considered to be statistically significant.

## Results

Of the 124 high-risk therapeutic devices approved by the FDA from 2016 to 2021, 2 (2%) were approved for use in children only, 23 (19%) for both children and adults, and 99 (80%) for adults only (Table 1). Among the 25 pediatric devices, 21 (84%) were approved via the Premarket Approval pathway and 4 (16%) through the Humanitarian Device Exemption program. Six pediatric devices (24%) were implantable compared with 66 of 99 adult devices (67%; *P* < .001). Fifteen pediatric devices (60%) were cardiovascular devices, which included 11 defibrillators (44%).

**Table 1.** Characteristics of High-risk Therapeutic Devices

Characteristic	Devices, No. (%)		*P* value^a^
	Pediatric (n = 25)	Adult only (n = 99)	
Approval pathways			
Premarket Approval	21 (84)	94 (95)	.06
Humanitarian Device Exemption	4 (16)	5 (5)	
Expedited review	4 (16)	8 (8)	.23
Life-sustaining	3 (12)	27 (27)	.11
Implantable	6 (24)	66 (67)	<.001
Approved pediatric ages			
0-17 y	11 (44)	NA	NA
Subgroup between 0 and 17 y	11 (44)		
Age not specified	3 (12)		
Therapeutic area			
Cardiovascular	15 (60)	46 (46)	.26
Endocrinology	3 (12)	2 (2)	
Orthopedics	3 (12)	10 (10)	
Ophthalmology	2 (8)	8 (8)	
Ear, nose, and throat	1 (4)	1 (1)	
Gastrointestinal	1 (4)	7 (7)	
Neurology	0	8 (8)	
Neurovascular	0	5 (5)	
General and plastic surgery	0	3 (3)	
Pulmonology	0	5 (5)	
Genitourinary	0	3 (3)	
Radiology	0	1 (1)	

Abbreviation: NA, not applicable.

^a^ *P* values calculated from χ^2^ tests.

Pediatric devices were approved based on 43 pivotal studies, of which 15 (35%) enrolled pediatric patients (Table 2). This total corresponded to 15 of 25 pediatric devices (60%) with pivotal studies that included children. Pivotal studies for pediatric devices enrolled a median (IQR) of 14 (7-49) children per pediatric device. By contrast, adult devices enrolled a median (IQR) of 265 (152-376) participants per device.

**Table 2.** Pivotal Studies and US Food and Drug Administration–Mandated Postapproval Studies for High-risk Therapeutic Devices

Characteristic	No./total No. (%)		*P* value^a^
	Pivotal studies for pediatric devices (n = 43)	Pivotal studies for adult only devices (n = 114)	
Devices with pivotal studies enrolling children	15/25 (60)	2/99 (2)	<.001
Participant enrollment, median (IQR)^b^			
No. of total participants enrolled per device	124 (78-184)	265 (152-376)	<.001
No. of pediatric participants enrolled per device	14 (7-49)	0	<.001
Study centers			
Multicenter	34 (79)	110 (96)	.001
Single center	2 (5)	2 (2)	
Not specified	7 (16)	2 (2)	
Randomization			
Randomized	21 (49)	56 (49)	.97
Nonrandomized	22 (51)	58 (51)	
Blinding			
Double-blind	11 (26)	15 (13)	<.001
Single blind	0	15 (13)	
Open label	24 (56)	84 (74)	
Not specified	8 (19)	0	
Comparator type			
Active comparator	19 (44)	51 (45)	.14
Placebo or sham	0	8 (7)	
Historical	0	4 (4)	
None	24 (56)	51 (45)	
End point type			
Clinical outcome	6 (14)	58 (51)	<.001
Surrogate marker	37 (86)	56 (49)	
Postapproval studies			
No. of postapproval studies	14/118 (12)	104/118 (88)	NA
Devices with postapproval study requirements	10/25 (40)	67/99 (68)	<.001
Postapproval studies with children in enrollment criteria	14/14 (100)	1/104 (1)	<.001
Status of postapproval studies			
Completed	1/14 (7)	14/104 (13)	.36^c^
Ongoing (progress adequate)	5/14 (36)	44/104 (42)	
Ongoing (progress inadequate)	3/14 (21)	6/104 (6)	
Terminated, other, or pending	5/14 (36)	40/104 (38)	

Abbreviation: NA, not applicable

^a^ *P* values calculated from χ^2^ tests for categorical variables and the Mood median test for medians.

^b^ For 7 of the 157 pivotal studies, the number of pediatric participants was not reported, and summary statistics were used to estimate the maximum number of pediatric patients that could have been enrolled.

^c^ *P* value for comparison of completed and ongoing (progress adequate) studies vs all others.

Postapproval studies were required for 10 of 25 pediatric devices (40%) compared with 67 of 99 adult devices (68%; *P* < .001). Among the 14 postapproval studies for pediatric devices, after a median (IQR) follow-up of 2.8 (2.3-4.0) years since device approval, 5 studies (36%) were progressing adequately and 1 (7%) had been completed.

## Discussion

Findings of this cohort study of high-risk therapeutic devices approved by the FDA from 2016 to 2021 suggest that, despite programs to increase pediatric medical device development, few devices are evaluated and approved for pediatric populations compared with adults. Furthermore, most pediatric devices are studied only in adult populations or in small numbers of pediatric patients. Postapproval studies could increase safety and effectiveness data for devices with limited premarket pediatric data or support labeling expansion of adult devices to pediatric patients. However, less than half of devices had FDA-mandated postapproval studies that enrolled children, and only 1 such study had been completed.

Limitations of the study include potential lack of generalizability to low- and moderate-risk devices. The findings support the critical need for new policies and further collaboration among regulators, academia, and industry to advance pediatric device development and address unmet device requirements in children.

## Notes

Supplement. **eTable.** High-risk Therapeutic Devices Approved by the FDA for Use in Children, 2016-2021
