# Supplementary material for: High-risk Therapeutic Devices Approved by the US Food and Drug Administration for Use in Children and Adolescents From 2016 to 2021
Source: JAMA Pediatr. 2022 Nov 7;177(1):98–100. doi: 10.1001/jamapediatrics.2022.4131 (PMC9641589; doi:10.1001/jamapediatrics.2022.4131)
Supplement: Supplement. — eTable. High-risk Therapeutic Devices Approved by the FDA for Use in Children, 2016-2021 [file jamapediatr-e224131-s001.pdf]

# Supplementary Online Content

Pathak K, Narang C, Hwang TJ, Espinoza JC, Bourgeois FT. High-risk therapeutic devices approved by the US Food and Drug Administration for use in children and adolescents from 2016 to 2021. *JAMA Pediatr*. Published online November 7, 2022. doi:10.1001/jamapediatrics.2022.4131

**eTable.** High-risk Therapeutic Devices Approved by the FDA for Use in Children, 2016-2021

This supplementary material has been provided by the authors to give readers additional information about their work.

© 2022 American Medical Association. All rights reserved.

**eTable.** High-risk Therapeutic Devices Approved by the FDA for Use in Children, 2016-2021

| Device Name | Device Type | Therapeutic Area |
|---|---|---|
| The 670G System | Insulin pump | Endocrinology |
| ZOLL® AED Plus® and Fully Automatic AED Plus® | External defibrillator | Cardiovascular |
| ZOLL® X Series®, R Series®, Propaq® MD, AED Pro®, and AED 3 BLS® | External defibrillator | Cardiovascular |
| HeartSine samaritan® PAD 350P (SAM 350P) HeartSine samaritan® PAD 360P (SAM 360P) HeartSine samaritan® PAD 450P (SAM 450P) Pad-Pak-01, Pad-Pak-02, and Pad-Pak-07 accessories Saver EVO® software version 1.4.0 | External defibrillator | Cardiovascular |
| LIFEPAK CR® Plus Defibrillator, LIFEPAK EXPRESS® Defibrillator, and CHARGE-PAK Battery Charger | External defibrillator | Cardiovascular |
| Flourish™ Pediatric Esophageal Atresia Device | Esophageal atresia device | Gastrointestinal |
| LIFEPAK® CR2 Defibrillator | External defibrillator | Cardiovascular |
| CustomFlex™ Artificial Iris | Artificial iris | Ophthalmology |
| Guardian Connect System | Glucose monitor | Endocrinology |
| t:slim X2 Insulin Pump with Basal-IQ Technology | Insulin pump | Endocrinology |
| Lifeline/ReviveR ECG and DDU Automated Defibrillators | External defibrillator | Cardiovascular |
| Cardiac Science Powerheart® AED G3 Pro | External defibrillator | Cardiovascular |
| Cardiac Science Powerheart® AED G3, G3 Plus, and G5 | External defibrillator | Cardiovascular |
| Minimally Invasive Deformity Correction (MID-C) System | Orthopedic device | Orthopedics |
| HeartStart OnSite Defibrillator (Model M5066A), HeartStart Home Defibrillator (Model M5068A), Primary Battery (Model M5070A), SMART Pads Cartridges (Adult Model M5071A) and Infant/Child (Model M5072A) | External defibrillator | Cardiovascular |
| Tula® System | Tympanostomy tube delivery system | Ear, nose and throat |
| MiSight 1 Day (omafilcon A) Soft (Hydrophilic) Contact Lenses for Daily Wear | Contact lens | Ophthalmology |
| The Tether™ - Vertebral Body Tethering System | Orthopedic device | Orthopedics |
| Kendall™ Multifunction Defibrillation Electrodes | Defibrillator components | Cardiovascular |
| HeartStart FRx Defibrillator (861304), Primary Battery (Model M5070A), Aviation FRx Battery (989803139301), SMART Pads II | External defibrillator | Cardiovascular |

© 2022 American Medical Association. All rights reserved.

| | | |
|---|---|---|
| (Model 989803139261), and Infant/Child Key (Model 989803139311) | | |
| Philips HeartStart FR3 Defibrillator, Primary Battery, Rechargeable Battery, Charger for the Rechargeable Battery, SmartPads III, DP pads, and Pediatric Key | External defibrillator | Cardiovascular |
| Sonalleve MR-HIFU | Ablation system for osteoid osteomas | Orthopedics |
| Edwards SAPIEN 3 Transcatheter Heart Valve System with Edwards Commander Delivery System | Heart valve | Cardiovascular |
| Medtronic Harmony Transcatheter Pulmonary Valve (TPV) System | Heart valve | Cardiovascular |
| ConMed PadPro Multifunction Electrodes, ConMed PadPro Multifunction Electrode Adapters | Defibrillator components | Cardiovascular |

© 2022 American Medical Association. All rights reserved.
